# Treatment of Small-Pox

**Published:** 1872-03

**Authors:** 


### The Treatment of Small-Pox.

Dr. Alexander Collie, the resident medical officer at the Homerton Fever Hospital, says that treatment in the mild variety of variola is unnecessary, and in the black small-pox useless. In the confluent form, however, treatment is of the greatest importance, and the result of the case will sometimes be determined by it. The room in which the patient is placed should be thoroughly ventilated, the windows being kept open even in winter. If possible there should be two beds in the room, in order that he may be changed from one to the other. He should be allowed a highly nutritious diet, consisting of milk, beef-tea, eggs beaten up with whiskey, tapioca. Cold water will be found most efficacious in relieving thirst, and the prejudice of the patient's friends should not be allowed to interfere with its administration. Effervescing drinks and lemonade may also be allowed. For heat of skin the patient may be sponged with cold water two or three times daily. If there be much restlessness or sleeplessness, the following, repeated in half an hour if needed, will be found of great service: Tincture of opium, 15 minims; spirit of ether, 15 minims; camphor water, 1 ounce; and if this fails stimulants may be given. For the soreness of throat, oleaginous of mucilaginous drinks may be given, and the following has also been found beneficial: Tincture of iron and glycerine, of each 39 minims three times a day. If laryngitis occur, a large linseed poultice should be applied round the throat, and the temperature of the room should be raised, and rendered moist by means of steam. All depressing remedies should be avoided, and tracheotomy should be performed whenever there is much interference with the respiration. If the patient becomes delirious, it is of the utmost importance that he should be treated with patience, gentleness, and firmness, and that no measures of restraint should be employed. Nothing has been found absolutely preventative of pitting. Common olive-oil may be used for this purpose in preference to applications which are more or less irritating. If diarrhœa occur, a mixture containing laudnum and sulphuric acid may be given.

In regard to the time when a small-pox patient may be considered free of danger to his neighbors, Dr. Collie says that this cannot be until all the products of disease are removed from his body, and until he presents all the ordinary indications of health, such as a normal temperature, a quiet pulse, a clean tongue, a clear mind, etc.—*Philadelphia Medical Times—New Remedies.*

